# A Nutraceutical Product Based on a Mixture of Algae and Extra Virgin Olive Oils and Olive Leaf Extract Attenuates Sepsis-Induced Cardiovascular and Muscle Alterations in Rats

**DOI:** 10.3389/fnut.2022.918841

**Published:** 2022-06-20

**Authors:** Daniel González-Hedström, Álvaro Moreno-Rupérez, María de la Fuente-Fernández, Mario de la Fuente-Muñoz, Marta Román-Carmena, Sara Amor, Ángel Luís García-Villalón, Asunción López-Calderón, Ana Isabel Martín, Teresa Priego, Miriam Granado

**Affiliations:** ^1^Departamento de Fisiología, Facultad de Medicina, Universidad Autónoma de Madrid, Madrid, Spain; ^2^R&D Department, Pharmactive Biotech Products S.L.U., Alcobendas, Madrid, Spain; ^3^Departamento de Fisiología, Facultad de Medicina, Universidad Complutense de Madrid, Madrid, Spain; ^4^CIBER Fisiopatología de la Obesidad y Nutrición, Instituto de Salud Carlos III, Madrid, Spain

**Keywords:** sepsis, nutraceutical, omega-3, extra virgin olive oil, olive leaf extract, cardiovascular, muscle wasting

## Abstract

Nutraceuticals are products of natural origin widely used for the treatment and/or prevention of some chronic diseases that are highly prevalent in Western countries, such as obesity or type II diabetes, among others. However, its possible use in the prevention of acute diseases that can put life at risk has been poorly studied. Sepsis is an acute condition that causes cardiovascular and skeletal muscle damage due to a systemic inflammatory state. The aim of this work was to evaluate the possible beneficial effect of a new nutraceutical based on a mixture of algae oil (AO) and extra virgin olive oil (EVOO) supplemented with an olive leaf extract (OLE) in the prevention of cardiovascular alterations and skeletal muscle disorders induced by sepsis in rats. For this purpose, male Wistar rats were treated with the nutraceutical or with water p.o. for 3 weeks and after the treatment they were injected with 1mg/kg LPS twice (12 and 4 h before sacrifice). Pretreatment with the nutraceutical prevented the LPS-induced decrease in cardiac contractility before and after the hearts were subjected to ischemia-reperfusion. At the vascular level, supplementation with the nutraceutical did not prevent hypotension in septic animals, but it attenuated endothelial dysfunction and the increased response of aortic rings to the vasoconstrictors norepinephrine and angiotensin-II induced by LPS. The beneficial effects on cardiovascular function were associated with an increased expression of the antioxidant enzymes SOD-1 and GSR in cardiac tissue and SOD-1 and Alox-5 in arterial tissue. In skeletal muscle, nutraceutical pretreatment prevented LPS-induced muscle proteolysis and autophagy and significantly increased protein synthesis as demonstrated by decreased expression of MURF-1, atrogin-1, LC3b and increased MCH-I and MCH -IIa in gastrocnemius muscle. These effects were associated with a decrease in the expression of TNFα, HDAC4 and myogenin. In conclusion, treatment with a new nutraceutical based on a mixture of AO and EVOO supplemented with OLE is useful to prevent cardiovascular and muscular changes induced by sepsis in rats. Thus, supplementation with this nutraceutical may constitute an interesting strategy to reduce the severity and mortality risk in septic patients.

## Introduction

Nutraceuticals are products of natural origin with active biological properties and defined beneficial health effects. The term nutraceutical encompasses a broad spectrum of commercially available products, including plant extracts, food products or dietary supplements such as herbs, enzymes, aminoacids, vitamins or minerals (1). In the last decades the use of nutraceutical products has spread worldwide, especially in some Western countries where more than 50% of the adult population recognizes to consume them regularly (2). However, despite their wide use for the treatment and/or prevention of chronic diseases (3), their preventive or therapeutical use in the context of acute life-threatening conditions is more limited.

Sepsis is a critical condition caused by an altered host response to infection. It results in the activation of intracellular mechanisms that promote the release of several inflammatory mediators which produce a systemic inflammatory syndrome leading to multiple organ failure (4). Indeed, septic shock is considered the main cause of death in intensive care units with a mortality rate ranging between 15 and 56% (5). In the last decades, the incidence of severe sepsis has steadily increased to a rate of 1.5% per annum (6), being considered a major health problem that affects millions of individuals worldwide (7) and the 10th most common cause of death in the United States (8). The World Health Organization has recently estimated 30 million cases of sepsis, 19.4 million by severe sepsis and 6 million deaths per year in the world (9).

A typical characteristic of septic shock and sepsis-induced multiorgan failure is the presence of cardiovascular dysfunction (10). Indeed, the mortality rate of septic patients with cardiovascular affection rises to 70–90% compared to 20% in septic patients without cardiovascular impairment (11).

Although the pathophysiology of sepsis is complex, one of the main cardiovascular alterations that contribute to the sepsis-induced multiorgan failure is reduced systemic vascular resistance (12) that is produced, at least in part, by the release of proinflammatory cytokines, nitric oxide (NO) and reactive oxygen species (ROS) into the blood by endothelial cells producing, among other consequences, hyporesponsiveness to vasoconstrictors and hypotension (13). Sepsis-induced organ dysfunction is also associated with endothelial barrier disruption which alters nutrient trafficking, cellular oxygenation, vascular tone and vascular permeability leading to microvascular destabilization and vascular leakage (14). In addition to impairment of endothelial function, vascular smooth muscle cells (VSMCs) also contribute to vascular dysfunction through the activation of toll-like receptor 4 (TLR4) which is also involved in the sepsis-induced hyporesponsiveness to vasoconstrictors (15, 16).

Studies in humans (17, 18) and in experimental animals (19–21) demonstrate that septic patients also suffer important cardiac alterations such as myocardial depression characterized by decreased contractility and impaired myocardial compliance. These alterations lead to impaired left ventricular systolic and diastolic function with reductions in stroke volume and ejection fraction (22). Several mechanisms have been proposed for the sepsis-induced myocardial dysfunction including the overexpression of adhesion molecules such as intercellular adhesion molecule-1 (ICAM-1) and vascular cell adhesion molecule-1 (VCAM-1) and the production and release of several substances such as cytokines, prostanoids, NO or endothelin-1 (ET-1) (23). Other factors that contribute to sepsis-induced myocardial impairment are altered flow autoregulation and/or disturbed oxygen utilization (24), as well as metabolic alterations such as decreased free fatty acid extraction, decreased glucose uptake or increased lactate extraction (25, 26). These alterations may increase the risk of suffering myocardial ischemia, especially in septic patients with coexistent or undiagnosed coronary artery disease (23). The coronary blood flow is reported to be increased in septic patients (27) which excludes the theory of global ischemia as a main contributor to myocardial damage. However, myocardial microcirculation undergoes major changes during sepsis that include endothelial disruption and blood flow maldistribution leading to heterogeneous cardiac microvascular blood flow (28). In addition, septic condition is reported to affect the recovery of cardiac function after ischemia-reperfusion (29, 30).

Sepsis also promotes skeletal muscle wasting dealing, in some cases, to a persistent myopathy characterized by atrophy and chronic weakness due to the inability to repair or regenerate dysfunctional myofibers (31). Skeletal muscle loss is associated both with decreased protein synthesis and increased protein degradation (32–35). Proteolytic activity is enhanced by overproduction of cytokines (36), excessive free radical generation (37) and changes in neuroendocrine factors (38). Sepsis also affects the expression of local factors involved in protein synthesis, such as IGF-1 (39), or markers involved in protein degradation, such as the atrogenes MURF-1 or atrogin-1 (40). Moreover histone deacetylases (HDAC) are reported to play an important role in muscle atrophy in different contexts such as denervation (41) or sarcopenia (42). Previous studies have reported that myogenin activation mediates the effects of HDAC4 on muscle wasting during prolonged muscle immobilization (43) or denervation (44). Therefore, the HDAC-4-myogenin axis seems to have an important regulatory role in the mechanisms of muscle disease and regeneration.

Current treatment of sepsis mainly relies on the timely and appropriate administration of supportive therapies and antibiotics. However, little is known about the possible use of nutraceuticals as a preventive strategy to ameliorate the sepsis-induced muscular and cardiovascular alterations to reduce morbidity and mortality.

Polyunsaturated Omega 3 fatty acids (ω3-PUFAs) are essential fatty acids with anti-inflammatory effects that are reported to improve cellular immune function in patients with sepsis (45, 46) and to reduce sepsis-induced mortality (47), possibly through increased production of pro-resolvin molecules like maresins, resolvins and protectins (48, 49). A recent study has reported that, in addition to ω3-PUFAs, olive tree derived products such as extra virgin olive oil and olive leaf extracts are also beneficial to prevent sepsis-induced inflammation and to increase the survival rate (50). Although there is some evidence about the therapeutic effect of these products, very little is known about their possible preventive effects reducing severe condition in septic patients. Thus, the aim of the present study is to analyze the possible beneficial effects of a new nutraceutical product based on a mixture of extra virgin olive oil (EVOO) and algae oil rich in ω3-PUFAs plus olive leaf extract in the prevention of the cardiovascular and muscle alterations associated with sepsis.

## Materials and Methods

### Animals

Twenty-four 3 months old male Wistar rats were housed under controlled conditions of temperature (22–24°C) and humidity (50–60%) with free access to standard chow and water. All the experiments were performed following the European Union Legislation and with the approval of the Animal Care Committee of the *Universidad Autónoma de Madrid* and the Autonomic Government of Madrid (PROEX 048/18).

### Treatments

A Cornicabra variety of EVOO with 80% of oleic acid and 60 mg/g of secoiridoids was obtained from Aceites Toledo S.A. (Los Yébenes, Spain). ω3-PUFA rich algae oil (AO) with 35% of docosahexaenoic acid (DHA), 20% of eicosapentaenoic acid (EPA) and 5% of docosapentaenoic acid (DPA) was obtained from DSM (Heerlen, Netherlands). Olive (*Olea europaea*) leaf extract (OLE) was provided by Pharmactive Biotech Products S.L.U. (Madrid, Spain). It was standardized to 1 mg/g of luteolin-7-O-glucoside by HPLC and to 30% of ortho-diphenols by UV/Vis. and stored in darkness until its addition into the feeding bottles.

During 21 days, rats were administered once daily by oral gavage either with 2.5 mL/Kg of tap water (Control; *n* = 8) or with 2.5 mL/kg of a mixture of 75% of EVOO and 25% of AO, and 100 mg/Kg of OLE dissolved in the drinking water. Over the three-week treatment, body weight and food and water intake were checked daily and weekly, respectively. The OLE dosage was adjusted to the liquid intake and to the animal’s body weights every 3 days. The proportion between AO and EVOO was chosen based on a previous study that demonstrates the increased stability of *n*-3 PUFAs when both oils were mixed in a proportion 75:25 (*w*/w) (51). Likewise, the duration of the intervention (21 days) and the daily dosages of AO: EVOO (2.5 mL/kg) and OLE (100 mg/kg) were chosen based on the positive effects found both in metabolic and cardiovascular function on previous studies with aged rats (52–54).

Twenty-one days after the initiation of the treatment, lipopolysaccharide (LPS; 1 mg/kg) was administered intraperitoneally twice (12 and 4 h before sacrifice) to half of the rats administered with vehicle (LPS; *n* = 8) and to the rats treated with the nutraceutical (Nutraceutical + LPS; *n* = 8). 1ml/kg of saline solution was administered intraperitoneally as vehicle following the same protocol (Control; *n* = 8).

All animals were killed by decapitation under an overdose of sodium pentobarbital (100 mg/kg). Serum was obtained from centrifuged trunk blood (20 min at 3000 rpm). The left gastrocnemius muscle and the spleen were immediately removed, weighed, and stored at −80°C for further analysis.

### Mean Arterial Pressure Measurement

After LPS treatment, mean arterial blood pressure (MAP) was measured by tail-cuff plethysmography as previously described (55). Five to six measurements were taken per animal to obtain the average.

### Serum Measurements

Glycaemia and the serum levels of metabolic hormones and lipids were assessed after 14 days of treatment and at sacrifice.

Glycaemia was measured using the glucometer Glucocard G (Arkray Factory, Inc.; Shiga, Japan). Insulin, leptin, and adiponectin serum levels were determined by ELISA kits according to manufacturer’s instructions (Merck Millipore, Dramstadt, Germany). The Homeostatic Model Assessment of Insulin Resistance (HOMA-IR) index was calculated through the following formula: fasting glucose (mg/dL) × (fasting insulin (ng/mL)/405). Serum levels of total lipids, triglycerides, total cholesterol, and low-density lipoprotein (LDL) and high-density lipoprotein (HDL) cholesterol were determined using colorimetric assays from Spin React (Sant Esteve de Bas, Girona, Spain).

### Cardiac Function

Hearts were removed immediately after the euthanasia and mounted in a Langendorff perfusion system as previously described (56). Briefly, a lateral connection in the perfusion cannula was used to measure the coronary perfusion pressure. To measure left ventricular pressure, a latex balloon was inflated to a diastolic pressure of 5–10 mm Hg. Both perfusion cannula and latex balloon were connected to Statham transducers (Statham Instruments, Los Angeles, CA, United States). The first derivative of the left ventricular pressure curve (dP/dt) was calculated through the left ventricular pressure values to get an index of heart contractility and heart rate. These parameters were recorded on a computer using the PowerLab/8e data acquisition system (ADInstruments, Colorado Springs, CO, United States). Flow perfusion was stopped for 30 min to induce a global ischemia after an equilibration period with constant flow perfusion of other 30 min. Afterward, hearts were re-perfused for 45 min. Hearts were collected and stored at −80°C for later analysis after ischemia-reperfusion (IR).

### Vascular Reactivity

To measure vascular reactivity, 2 mm of aorta segments were cut in sterile cold saline solution (NaCl 9 g/L) and mounted in an organ bath system as previously described (57). Briefly, two steel wires (100 μm) were passed through the lumen of the segments. One of the wires was fixed to a 4 mL organ bath chamber filled with modified Krebs-Henseleit solution (NaCl 115, KCl 4.6, KH_2_PO_4_ 1.2, MgSO_4_ 1.2, CaCl_2_ 2.5, NaHCO_3_ 25, glucose 11; mM) at 37°C and pH of 7.3–7.4 The other one was connected to a strain gauge for isometric tension recording using a PowerLab data acquisition system (AD Instruments; Colorado Springs, CO, United States). An optimal passive tension of 1 g was applied to each segment during an equilibration period 60–90 min. Afterward, segments were stimulated with 100 mM of KCl to measure their contraction capacity. Segments were discarded if they failed to contract at least 0.5 g to KCl.

Vasoconstriction response to cumulative doses of noradrenaline (10^–9^–10^–4^ M) and angiotensin-II (10^–11^–10^–6^ M) were measured in abdominal aortic segments. Results were expressed as percentage of the contraction to KCl 100 mM.

Vasodilator response to cumulative doses of acetylcholine (10^–9^–10^–4^ M) and sodium nitroprusside (10^–9^–10^–5^ M), and to insulin 10^–6^ M were measured in thoracic aortic segments precontracted with phenylephrine 10^–7^.^5^ M. Relaxation was expressed as a percentage of the final tone after sodium nitroprusside (10^–5^ M) stimulation.

All drugs were obtained from Sigma-Aldrich (St. Louis, MO, United States).

### RNA Extraction and Quantification

Tri-Reagent protocol was used to extract total RNA from heart, aorta and left gastrocnemius tissues (58). After RNA quantification using a Nanodrop 2000 (Thermo Fisher Scientifics, Hampton, NH, United States), cDNA was obtained from 1 g of total RNA using a high-capacity cDNA reverse transcription kit (Applied Biosystems; Foster City, CA, United States).

### Quantitative Real-Time Polymerase Chain Reaction

Assay-on-demand kits (Applied Biosystems, Foster City, CA, United States) were used to measure the gene expression of tumoral necrosis factor α (TNF-α), interleukins -1β (IL-1β), −6 (IL-6) and −10 (IL-10), glutathione peroxidase (GPx) and reductase (GSR), superoxide dismutase-1 (SOD-1) and lipoxygenase (Alox5) in myocardial, aortic and gastrocnemius tissues by quantitative real-time polymerase chain reaction (qPCR). The messenger ribonucleic acid (mRNA) levels of NADPH oxidases 1 (NOX-1) and 4 (NOX-4) were also determined in the heart and aorta, and mRNA levels of atrogin-1, Muscle RING-finger protein-1 (MuRF1), Microtubule-associated proteins 1A/1B light chain 3B (LC3b), Insulin growth factor 1 (IGF-1), IGF binding protein 3 (IGFBP3) and Myosin heavy chain isoform I and IIa (MHC-I and MHC-IIa) were determined in the gastrocnemius. Amplification was performed by using the TaqMan Universal PCR Master Mix (Applied Biosystems, Foster City, CA, United States) in a Step One machine (Applied Biosystems, Foster City, CA, United States). 18S and/or hypoxanthine guanine phosphoribosyl transferase (HPRT) were used as housekeeping genes and relative gene expression was determined by the ΔΔC_T_ method (59).

### Protein Analysis in Gastrocnemius Muscle by Western Blot

100 mg of left gastrocnemius muscle were homogenized in lysis buffer (Radioimmunoprecipitation Assay (RIPA) buffer, 10 μL/mg) in presence of a protease inhibitor cocktail (Phenylmethane sulfonyl fluoride 100 mM, sodium orthovanadate 12.5 mM and sodium deoxycholate 12.5 mM; Sigma-Aldrich, St. Louis, MO, United States). Total proteins were obtained on the supernatant after centrifugation at 13,000 rpm for 30 min at 4°C was and quantified by Bradford colorimetric assay (Sigma-Aldrich; St. Louis, MO, United States). 50 μg of the protein extracts were mixed (1:1) with Laemmli loading buffer (Bio-Rad; Madrid, Spain) and heated at 95°C for 5 min. Protein extracts were then resolved by electrophoresis on a polyacrylamide 8–12% gradient gels (Bio-Rad; Madrid, Spain) under reducing conditions. Afterward, proteins were transferred onto nitrocellulose membranes, using Ponceau-S staining to ensure optimal protein transference. Then, membranes were blocked with 0.1% Tween and 5% non-fat dry milk (Sigma-Aldrich; St. Louis, MO, United States) in Tris-buffered saline. Membranes were sequentially incubated overnight at 4°C with a primary antibodies against Histone deacetylase 4 (HDAC-4; antibody ID: 7628, 1:2000; Cell Signaling Technology; Danvers, MA, United States), myogenin (antibody ID: sc-12732, 1:500; Santa Cruz Biotechnology; Dallas, TX, United States) and microtubule-associated protein-1 light chain 3 (LC3b) A/B (D3U4C) XP (antibody ID: 12741, 1:1000; Cell Signaling Technology; Danvers, MA, United States). Before adding each primary antibody to the membranes stripping was performed to eliminate previous antibodies (Restore Western Blot Stripping Buffer, Thermo Scientific; Rockford, IL, United States). After incubation during 90 min with the appropriate secondary antibody conjugated to horseradish peroxidase (anti-rabbit IgG, GE Healthcare; Chicago, IL, United States), peroxidase activity was visualized by chemiluminescence using a PC-Image VGA24 (Thermo Scientific; Rockford, IL, United States) program for Windows.

### Statistical Analysis

All values are expressed as means ± standard error of the mean (SEM). One-way ANOVA followed by Bonferroni *post hoc* test was used for the statistical data analysis using GraphPad Prism 8.0 (San Diego, California, United States). A *p*-value of ≤ 0.05 was considered statistically significant.

## Results

### Effects of LPS and Supplementation With the Nutraceutical on Body and Organ Weights

The changes in body and organ weights after administration of saline or LPS to rats pretreated with vehicle or the nutraceutical for 3 weeks are shown in Table 1.

**TABLE 1:** Body weight gain, mean arterial pressure (MAP) and spleen and gastrocnemius weights of rats treated with vehicle, LPS or LPS + Nutraceutical.

	Control	LPS	Nutraceutical + LPS	C vs. L (*p*-value)	C vs. N + L (*p*-value)	L vs. N + L (*p*-value)
Δ Body weight (g)	2.9 ± 0.8	−17.8 ± 1.7	−10.9 ± 2.5	0.000	0.001	0.019
MAP (mm Hg)	122.1 ± 3.5	107.9 ± 3.0	104.7 ± 2.9	0.006	0.002	0.233
Spleen weight (mg/100 g bw)	180.1 ± 9.6	248.9 ± 10.4	204.8 ± 14.6	0.000	0.023	0.017

*Data are represented as mean value ± SEM; n = 8 samples/group.*

*C, control; L, LPS; N + L, nutraceutical + LPS.*

Supplementation with the nutraceutical for 3 weeks significantly attenuated LPS-induced body weight loss (*p* < 0.05). Likewise, pretreatment with the nutraceutical prevented LPS-induced increase in spleen weight (*p* < 0.05).

### Effects of LPS and Supplementation With the Nutraceutical on Myocardial Function

**FIGURE 1:**
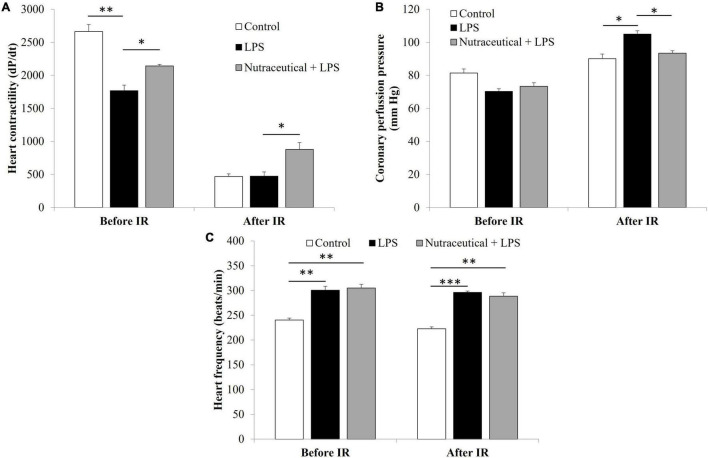
Heart contractility **(A)**, coronary perfusion pressure **(B)** and heart frequency before and after ischemia reperfusion (IR) of rats treated with vehicle, LPS or LPS + Nutraceutical. Values are represented as mean ± SEM. *p < 0.05; ^**^p < 0.01; ^***^p < 0.001.

Results of heart function in basal conditions and after coronary ischemia-reperfusion are shown in Figure 1.

In basal conditions, hearts from LPS-treated rats showed decreased contractility (Figure 1A, *p* < 0.001) and increased heart rate (Figure 1C, *p* < 0.01) compared to controls. Supplementation with the nutraceutical did not prevent the LPS-induced alterations on heart rate but it significantly attenuated the decrease on myocardial contractility (*p* < 0.05).

IR induced a decrease in heart contractility in all experimental groups (Figure 1A, *p* < 0.001) and a significant increase in coronary perfusion pressure (Figure 1B, *p* < 0.05) and heart rate in LPS injected rats treated with vehicle (Figure 1C, *p* < 0.01). Pretreatment with the nutraceutical for 3 weeks did not ameliorate the IR and LPS-induced changes on heart rate but it prevented the increase in coronary perfusion pressure and the decrease in dP/dt (*p* < 0.05 for both).

### mRNA Levels of Markers Related to Inflammation and Oxidative Stress in Myocardial Tissue

**FIGURE 2:**
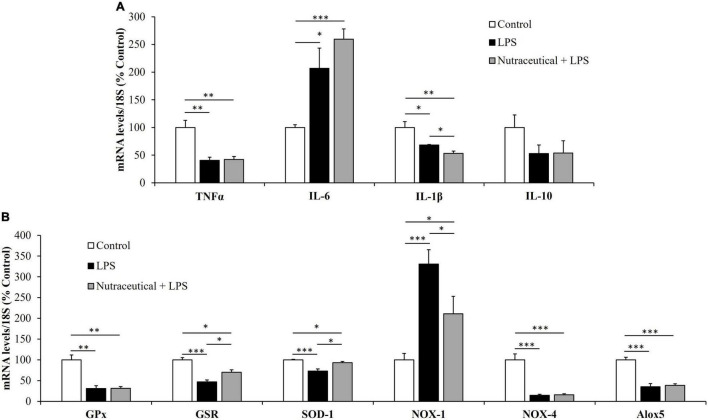
mRNA concentrations of tumor necrosis factor α, Interleukin 6, Interleukin 1β and Interleukin 10 **(A)** and glutathione peroxidase, glutathione reductase, super oxide dismutase 1, NADPH oxidase 1 and 4, and Lipoxygenase **(B)** in the heart of rats treated with vehicle, LPS or LPS + Nutraceutical. Values are represented as mean ± SEM. *p < 0.05; ^**^p < 0.01; ^***^p < 0.001.

The gene expression of markers related to inflammation and oxidative stress is shown in Figures 2A,B, respectively.

Sepsis induced a significant increase in the mRNA levels of the inflammatory marker IL-6 (*p* < 0.05) whereas the gene expression of TNF-α and IL-1β was reduced (*p* < 0.01 and *p* < 0.05, respectively). Likewise, LPS downregulated the mRNA levels of GPx (*p* < 0.01), GSR (*p* < 0.001), SOD-1 (*p* < 0.001), NOX-4 (*p* < 0.001) and Alox-5 (*p* < 0.001) and upregulated the gene expression of NOX-1 (*p* < 0.001).

Supplementation with the nutraceutical for 3 weeks did not prevent the LPS-induced changes in the gene expression of TNF-α, IL-6, IL-1β, GPx, NOX-4 and Alox5 but it attenuated the alterations in the mRNA levels of GSR, SOD-1 and NOX-1 (*p* < 0.05 for all). Moreover, pretreatment with the nutraceutical accentuated the decrease in IL-1β induced by LPS (*p* < 0.05). Finally, the gene expression of IL-10 was not modified either by LPS or by pretreatment with the nutraceutical.

### Vascular Response of Aorta Segments to the Vasoconstrictors Noradrenalin and Angiotensin II

**FIGURE 3:**
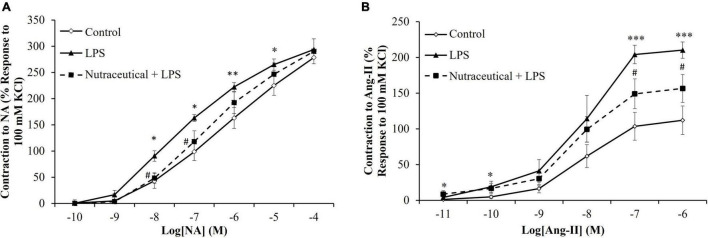
Contraction response of abdominal aortic segments to norepinephrine **(A)** and to angiotensin-II **(B)** of rats treated with vehicle, LPS or LPS + Nutraceutical. Values are represented as mean ± SEM. *p < 0.05 vs. Control; ^**^p < 0.01 vs. Control; ^***^p < 0.001 vs. Control; ^#^*p* < 0.05 vs. LPS.

LPS induced a significant increase in the vasoconstrictor response of aorta segments to NA (Figure 3A) at the concentrations of 10^–8^ M (*p* < 0.05), 10^–7^ M (*p* < 0.05), 10^–6^ M (*p* < 0.01) and 10^–5^ M (*p* < 0.05). Likewise, the contraction in response to AngII (Figure 3B) was significantly higher in aorta segments from LPS-injected animals compared to controls at the concentrations 10^–7^ M and 10^–6^ M (*p* < 0.001 for both).

Supplementation with the nutraceutical attenuated the LPS-increased contraction to NA at the dosages of 10^–8^ M and 10^–7^ M (*p* < 0.05 for both) and to AngII at the dosages of 10^–7^ M and 10^–6^ M (*p* < 0.05 for both).

### Vascular Response of Aorta Segments to the Vasodilators Acetylcholine, Sodium Nitroprusside, and Insulin

**FIGURE 4:**
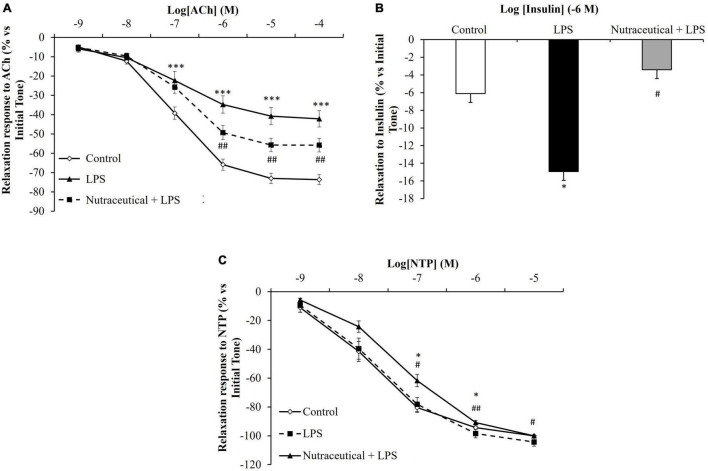
Relaxation response of thoracic aortic segments to acetylcholine **(A)**, to insulin **(B)** and to sodium nitroprusside **(C)** of rats treated with vehicle, LPS or LPS + Nutraceutical. Values are represented as mean ± SEM. *p < 0.05 vs. Control; ^***^p < 0.001 vs. Control; ^#^< 0.05 vs. LPS; ^##^*p* < 0.01 vs. LPS.

Aorta segments from septic rats showed decreased relaxation in response to Ach at the concentrations of 10^–7^ M to 10^–4^ M (Figure 4A, *p* < 0.001 for all) and this effect was attenuated by pretreatment with the nutraceutical at dosages of 10^–6^ M to 10^–4^ M (*p* < 0.001 for all). Likewise, supplementation with the nutraceutical prevented the LPS-induced increase relaxation of aorta segments in response to insulin 10^–7^ M (Figure 4B, *p* < 0.01). Finally, the relaxation of aortic rings to NTP was not modified in response to LPS but significantly reduced in aorta segments from septic rats pretreated with the nutraceutical at the dosages of 10^–7^ M to 10^–5^ M (Figure 4C, *p* < 0.05 for all).

### mRNA Levels of Markers Related to Inflammation and Oxidative Stress in Arterial Tissue

**FIGURE 5:**
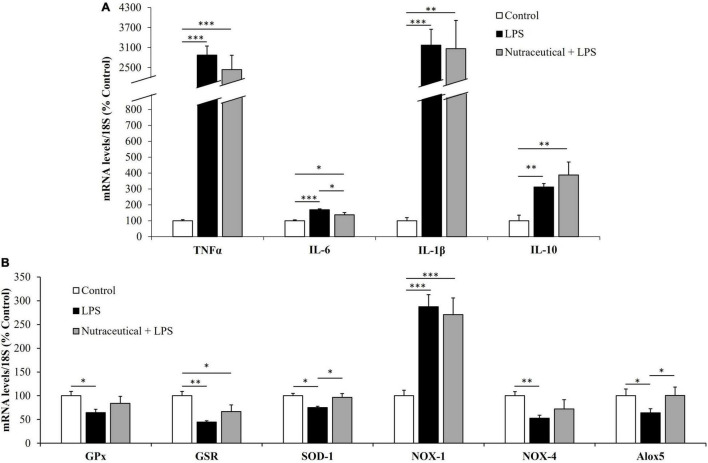
mRNA concentrations of tumor necrosis factor α, Interleukin 6, Interleukin 1β and Interleukin 10 **(A)** and Glutathione Peroxidase, Glutathione Reductase, Super Oxide Dismutase 1, NADPH oxidase 1 and 4, and Lipoxygenase **(B)** in the aorta of rats treated with vehicle, LPS or LPS + Nutraceutical. Values are represented as mean ± SEM. *p < 0.05; ^**^p < 0.01; ^***^p < 0.001.

The mRNA levels of inflammatory and oxidative stress markers in arterial tissue are shown in Figures 5A,B, respectively.

Sepsis induced an overexpression of the inflammatory mediators TNF-α, (*p* < 0.001) IL-6, (*p* < 0.001), IL-1β (*p* < 0.001) and IL-10 (*p* < 0.01). In addition, LPS downregulated the gene expression of GPx (*p* < 0.05), GSR (*p* < 0.01), SOD-1 (*p* < 0.05), NOX-4 (*p* < 0.01) and Alox5 (*p* < 0.05) and induced an overexpression of NOX-1 (*p* < 0.001). Pretreatment with the nutraceutical did not ameliorate the LPS induced alterations in the gene expression of TNF-α, IL-1β, IL-10, GPx, GSR and NOX-1 but it prevented the increase in IL-6 and the decrease in SOD-1 and Alox5 (*p* < 0.05 for all).

### Expression of Proteolytic Markers, Muscle Regulatory Factors and Myosin Heavy Chain in Skeletal Muscle

**FIGURE 6:**
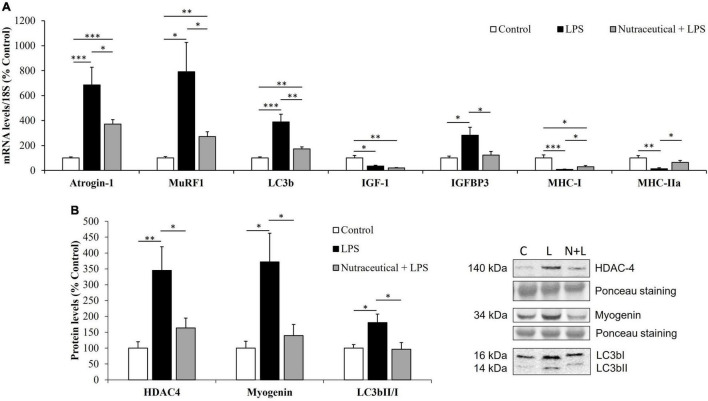
mRNA concentrations of Atrogin-1, Muscle RING-finger protein-1, Microtubule-associated proteins 1A/1B light chain 3B, Insulin growth factor 1, IGF binding protein 3 and Myosin heavy chain isoforms I and IIa **(A)** and protein levels of Histone deacetylase 4, Myogenin and LC3bII/I ratio **(B)** in the gastrocnemius of rats treated with vehicle, LPS or LPS + Nutraceutical. Values are represented as mean ± SEM. *p < 0.05; ^**^p < 0.01; ^***^p < 0.001. C, Control; L, LPS; N + L, Nutraceutical + LPS.

As shown in Figure 6A, pretreatment with the nutraceutical partially prevented the sepsis-induced upregulation (*p* < 0.001) in the mRNA levels of the ubiquitin ligases, atrogin-1 and MuRF1 (*p* < 0.05 for both) and the autophagy marker LC3b (*p* < 0.01) in gastrocnemius muscle. In addition, LPS also increased the lipidation of the LC3b protein, measured as the ratio of the two forms LC3B II/I in the rats pretreated with water (Figure 6B, *p* < 0.05), but not in those rats that received the nutraceutical pretreatment. Sepsis also increase HDAC4 (*p* < 0.05) and myogenin (*p* < 0.01) protein levels in gastrocnemius muscle and nutraceutical pretreatment prevented this increase (Figure 6B).

Figure 6A shows that sepsis was also associated with a significant decrease in the mRNA levels of IGF-1 (*p* < 0.05), MHC-I (*p* < 0.001) and MHC-IIA (*p* < 0.01) mRNA levels and with an overexpression of IGFBP-3 (*p* < 0.05) in gastrocnemius muscle. Pretreatment with the nutraceutical did not attenuate the effect of LPS on IGF-1 mRNA, but it prevented the LPS-induced changes in the gene expression of IGFBP-3, MHC-I and MHC-IIa (*p* < 0.05 for all).

### mRNA Levels of Markers Related to Inflammation and Oxidative Stress in Skeletal Muscle

**FIGURE 7:**
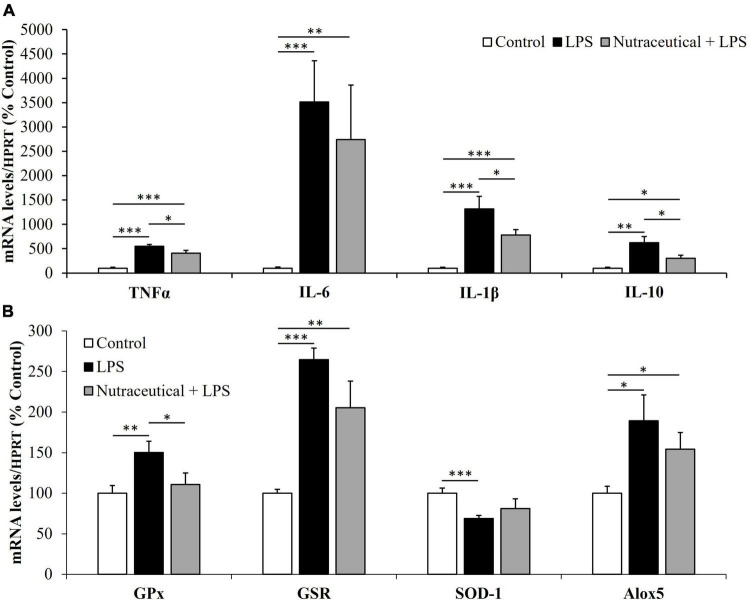
mRNA concentrations of tumor necrosis factor α, Interleukin 6, Interleukin 1β and Interleukin 10 **(A)** and Glutathione Peroxidase, Glutathione Reductase, Super Oxide Dismutase 1 and Lipoxygenase **(B)** in the gastrocnemius of rats treated with vehicle, LPS or LPS + Nutraceutical. Values are represented as mean ± SEM. *p < 0.05; ^**^p < 0.01; ^***^p < 0.001.

The mRNA levels of inflammatory and oxidative stress markers in gastrocnemius muscle are shown in Figures 7A,B, respectively.

Regardless of the pretreatment they received, sepsis equally increased the mRNA levels of TNF-α, IL-1, IL-6 (*p* < 0.001) and IL-10 (*p* < 0.01) in both groups of septic rats. However, the mRNA levels of TNF-α, IL-1 and IL-10 were significantly lower in the group of septic rats pretreated with the nutraceutical compared to untreated ones (*p* < 0.05).

Sepsis increased the expression of GPx (*p* < 0.01), GSR (*p* < 0.001) and Alox5 (*p* < 0.05) and decreased SOD-1 (*p* < 0.001) mRNA in the skeletal muscle (Figure 7B). Pretreatment with the nutraceutical prevented the effects of sepsis on GPx and Alox-5 (*p* < 0.05 for both), but not on GSR and SOD-1 mRNA levels.

### Serum Measurements Before LPS Administration

The serum levels of different markers related to adiposity, lipid profile and insulin sensitivity were measured after 14 days of treatment with the nutraceutical and are shown in Table 2.

**TABLE 2:** Glycemia, lipid profile, insulin, and HOMA-IR in the serum of rats treated with vehicle, LPS or LPS + Nutraceutical.

	Control	Nutraceutical	C vs. N (*p*-value)
Glycemia (mg/dl)	114.5 ± 5.1	68.5 ± 2.6	0.000
Insulin (ng/ml)	8.9 ± 1.0	11.5 ± 1.7	0.112
HOMA-IR index	2.5 ± 0.4	1.8 ± 0.3	0.045
Adiponectin	69.4 ± 3.1	85.7 ± 4.7	0.010
Leptin	5.9 ± 0.7	12.6 ± 2.4	0.006
Total lipids (mg/dl)	385.8 ± 38.3	334.5 ± 31.7	0.321
Triglycerides (mg/dl)	87.1 ± 9.2	120.3 ± 10.5	0.035
Total cholesterol (mg/dl)	101.8 ± 6.5	80.9 ± 5.5	0.041
LDL-cholesterol (mg/dl)	15.6 ± 2.7	6.7 ± 0.7	0.010
HDL-cholesterol (mg/dl)	6.8 ± 1.2	12.9 ± 1.2	0.004

*Data are represented as mean value ± SEM; n = 8 samples/group.*

*HDL, high density lipoprotein; HOMA-IR, Homeostatic Model Assessment of Insulin Resistance; LDL, low density lipoprotein; C, Control; N, nutraceutical.*

Supplementation with the nutraceutical for 2 weeks did not modify the circulating levels of total lipids and triglycerides but it significantly reduced the serum concentrations of total cholesterol (*p* < 0.05) and LDL-c (*p* < 0.01) and increased the circulating levels of HDL-c (*p* < 0.05). Moreover, pretreatment with the nutraceutical increased the serum concentrations of both leptin and adiponectin (*p* < 0.01 for both), and decreased glycaemia (*p* < 0.001) and HOMA-IR (*p* < 0.05). However, the circulating levels of insulin were unchanged between treated and untreated rats.

## Discussion

In this study we report for the first time the beneficial effects of pretreatment with a new nutraceutical product based on a mixture of algae and extra virgin olive oils and an olive leaf extract in the cardiovascular and skeletal muscle alterations induced by sepsis.

As stated above cardiovascular impairment is one of the most common features of septic shock and is present in most of the patients with fatal end (11). For this reason, it is necessary to search for new therapeutic and/or preventive agents that help to improve cardiovascular function and reduce the morbidity and mortality of patients suffering from this pathology.

As previously described, our results show that sepsis is associated with a significant increase in heart rate and a decrease in both coronary pressure and myocardial contractility (22–24, 60). The daily administration of the nutraceutical 3 weeks before sepsis induction did not affect neither the coronary pressure nor the heart rate in septic rats, but it prevented the LPS-induced decrease in myocardial contractility, both before and after ischemia-reperfusion. Moreover, supplementation with the nutraceutical also prevented the increase in the pressure of coronary arteries after IR in septic animals. These results agree with previous studies that have reported that supplementation with ω3-PUFAs improves cardiac function in an animal model of sepsis (61). However, supplementation with this nutraceutical to aged rats did not have a positive effect on cardiac function neither before nor after IR (unpublished data from our group). Thus, as this model was performed with young animals, it is possible that the beneficial effects of the nutraceutical on cardiac function are age dependent. The cardioprotective effects of the nutraceutical do not seem to be due to the amelioration of inflammation since no significant differences were found in the gene expression of several pro-inflammatory mediators in myocardial tissue between septic animals supplemented or not with the nutraceutical. On the contrary, septic rats pretreated with the nutraceutical showed increased gene expression of the antioxidant enzymes GSR and SOD-1 and decreased mRNA levels of the pro-oxidant enzyme NOX-1 in myocardial tissue, pointing that the myocardial beneficial effects are most likely due to reduced oxidative stress, as it has been previously described after supplementation with ω3-PUFAs in experimental models of sepsis (61, 62). Moreover, transgenic *fat-1* mice, which have increased endogenous production of ω3-PUFAs, are protected from the detrimental LPS-induced inflammation and oxidative stress and exhibit decreased mortality compared to wild-type mice (63).

Derivatives from the olive tree may also exert a positive impact on myocardial function in septic animals since both olive oil (64) and olive leaf extract (65, 66) are reported to exert direct cardioprotective effects that are attributable to some of their components such as oleuropein (67–69) and hydroxytyrosol (66, 70).

In addition to the cardioprotective effects, our results also show that pretreatment with the nutraceutical for 3 weeks prevents some of the LPS-induced alterations in vascular function possibly through decreased inflammation and oxidative stress in arterial tissue, as it has been previously described (52–54). These effects include an improvement in both endothelium dependent and endothelium independent relaxations of aortic rings, as well as attenuation of LPS-induced increased vasoconstriction in response to noradrenalin. These results are in agreement with previous studies from our group in which supplementation with the different ingredients of this nutraceutical product to aged rats for 3 weeks attenuated endothelial dysfunction and prevented the aging-induced increase in the vasoconstrictor response of aorta segments to NA, both when administered separately (52, 54) or in combination (53). Moreover, the increased response of aorta segments to AngII induced by LPS was attenuated in animals pretreated with the nutraceutical, with this fact possibly being related to the conversion of AngII into the vasodilator peptide angiotensin 1–7, (71, 72), as it is reported in *fat-1* mice (63). Another important finding of this work is that sepsis is associated with increased relaxation of aorta segments in response to insulin and that this effect is completely prevented by the treatment with the nutraceutical. Although the vasodilator effects of insulin are widely reported (73), to our knowledge, the increased relaxation of arterial segments in response to insulin after LPS administration had not been reported before. The physiological meaning of this increased relaxation requires further investigation, but, since endotoxemia is associated with insulin resistance in several tissues (74, 75), it may serve to increase glucose supply into the tissues. If this was the case, the normalization of this response in the animals supplemented with the nutraceutical may indicate improved insulin sensitivity, as it has been previously reported in aged rats (53).

Despite the beneficial effects on cardiac and vascular function, our results show that supplementation with the nutraceutical did not ameliorate LPS-induced reduction of mean arterial pressure. This result agrees with a previous study in which pretreatment with ω3-PUFAs exerted a cardioprotective effect but did not attenuate the LPS-induced hypotension (61).

In skeletal muscle, our results show that pretreatment with the nutraceutical ameliorates the stimulatory effect of sepsis on the gene expression of the proteolytic markers atrogin-1 and MuRF1, as well as on the mRNA levels of LC3b, myogenin and HDAC4. In addition to atrogin-1 and MuRF1 overexpression, an increase in muscle HDAC4 and myogenin has been recently reported in sepsis (76), and in other conditions that induce muscle atrophy such as neurological disease (77), disuse (78) and aging (53). Furthermore, HDAC4 inhibition is reported to decrease denervation-induced muscle atrophy through decreased expression of muscle specific E3 ubiquitin ligases atrogin-1 and MuRF1 and autophagy related proteins (79). In this regard, we have previously reported that the treatment with this nutraceutical was also able to prevent the activation of the HDAC4-myogenin axis and the skeletal muscle loss induced by aging in rats (53). Likewise, extra virgin olive oil enriched diet down-regulates the expression of the autophagy marker LC3b in an amyotrophic lateral sclerosis mice model, together with higher survival and better motor performance (80). Therefore, the beneficial effect of the nutraceutical may be secondary to its effects preventing LPS-induced increase in HDAC4, myogenin and LC3b expression.

The protective effect of nutraceutical pretreatment on skeletal muscle in septic rats was also observed in anabolic pathways, since the nutraceutical prevented the down regulation of the contractile protein mRNA MHC-I and MHC-IIa induced by LPS. In addition, although the nutraceutical was not able to prevent the decrease in IGF-1 mRNA, it prevented the LPS-induced increased in IGFBP-3 expression. The fact that IGFB-3 has been reported to increase muscle proteolysis and myoblast proliferation and differentiation (81) suggests that the nutraceutical effect decreasing IGFBP-3 levels in the gastrocnemius contributes to preserve skeletal muscle mass.

Another factor that plays an essential role in muscle atrophy is TNFα (82), which is markedly upregulated in the gastrocnemius of untreated septic rats. Nutraceutical pretreatment attenuated LPS-induced increase in both TNFα and IL-1β mRNA levels in skeletal muscle. Thus, the protective effect of the nutraceutical pretreatment preventing proteolysis and promoting contractile protein synthesis in the skeletal muscle can be secondary to its anti-inflammatory properties.

Since both cardiovascular and skeletal muscle function are closely affected by the metabolic state, the beneficial effects of supplementation with this new nutraceutical on skeletal muscle and the cardiovascular system could be related to its positive effects on the circulating levels of different serum parameters related to both lipid profile and insulin sensitivity. In this regard, our results show that supplementation with the nutraceutical for 2 weeks significantly increased the serum levels of HDL-cholesterol and reduced the circulating levels of both total and LDL-cholesterol. These results agree with previous studies of our group in which supplementation with the different ingredients of this nutraceutical, administered either alone (52, 54) or together (53), attenuates some the aging-induced alterations in the lipid profile, such as the decrease in the circulating levels of LDL-c or the increase in the serum concentrations of HDL-c. Our results also show a positive effect of the nutraceutical decreasing glycaemia and the HOMA-IR, an index of peripheral insulin sensitivity, after 2 weeks of supplementation. Likewise, treatment with the same nutraceutical for 3 weeks improves insulin sensitivity in aged rats through a decrease in the HOMA-IR and the circulating levels on insulin. This effect is produced by both the oil mixture (52) and the olive leaf extract (54). However, only the co-administration of both treatments increases the circulating concentrations of adiponectin, which reveals a synergistic effect of both ingredients (53).

In conclusion, supplementation with a new nutraceutical product based on a mixture of extra virgin olive and algae oils and an olive leaf extract exerts a preventive effect ameliorating the alterations in skeletal muscle and cardiovascular function associated with sepsis through its anti-inflammatory and antioxidant effects. Thus, supplementation with this nutraceutical may be useful to decrease the morbidity and mortality in septic patients.

## Data Availability Statement

The original contributions presented in this study are included in the article/supplementary material, further inquiries can be directed to the corresponding author/s.

## Ethics Statement

The animal study was reviewed and approved by the Animal Care Committee of the Universidad Autónoma de Madrid and the Autonomic Government of Madrid (PROEX 048/18).

## Author Contributions

MG designed the study. DG-H, ÁM-R, MF-F, MF-M, MR-C, SA, ÁG-V, AL-C, AI, TP, and MG conducted the research and analyzed the data, and performed the statistical analysis. DG-H and MG wrote the manuscript. MG had primary responsibility for final content. All authors read and approved the final manuscript.

## Conflict of Interest

DG-H was employed by Pharmactive Biotech Products S.L.U. The remaining authors declare that the research was conducted in the absence of any commercial or financial relationships that could be construed as a potential conflict of interest. The authors declare that this study was done in colaboration with the pharmaceutical company Pharmactive Biotech Products S.L.U. The company was not involved in the study design, collection, analysis, interpretation of data, the writing of this article or the decision to submit it for publication.

## Publisher’s Note

All claims expressed in this article are solely those of the authors and do not necessarily represent those of their affiliated organizations, or those of the publisher, the editors and the reviewers. Any product that may be evaluated in this article, or claim that may be made by its manufacturer, is not guaranteed or endorsed by the publisher.
